# Psychological aspects of bracing in scoliosis in relation to age and duration of brace-wear

**DOI:** 10.1186/1748-7161-8-S2-O42

**Published:** 2013-09-18

**Authors:** Nina Byskosh

**Affiliations:** 1 Arlington Heights, IL USA

## Background

Numerous questionnaires, including SRS-22, SRS-23, SRS-24 and BSSQ, were created to evaluate the psychological aspects of bracing young individuals. The study of the psychological impacts of bracing has proven beneficial in the comprehensive treatment plan for the young patient. 

## Purpose

The purpose of this study was to use the NBSQ questionnaire to evaluate the emotional aspects, self-image, self-esteem, social support and coping skills of individuals braced for scoliosis.

## Methods

The NBSQ questionnaire, which consists of 31 questions, was posted online to be completed by those who have experienced bracing for scoliosis. The questionnaire was completed by 139 people from 10 countries. Subjects were divided into three age groups: pre-adolescents (8-12), adolescents (13-17) and late-adolescents (18-21). A total of 113 completed questionnaires were used and evaluated according to their emotional aspects, self-image, self-esteem, social support and coping skills. In addition, demographics were incorporated into the study. These groups were further analyzed according to hours of brace-wear per day. Data was analyzed using means, standard deviations and student’s t-test. 

## Results

Differences among various age groups were found, and individuals who wore a brace for more than 12 hours per day showed more signs of emotional distress than those who wore a brace for less than 12 hours per day. Additionally, individuals who wore the brace for more than 12 hours per day revealed a significant lifestyle change, which was indicated in the pre-adolescent group (p- value: 0.01038). Differences in self-image were observed in all age groups, including a significant difference in pre-adolescents (p- value: 0.04615). In relation to social support, it was observed that parents (91.5%) were the main source of support, followed by doctors (62.4%), friends (60.1%) and siblings (25.5%). 

## Conclusions and discussion

The results observed support the hypothesis that bracing affects patients psychologically. Significant differences were observed between various age groups and the total amount of brace-wear per day. 

## References

[B1] Tomaszewski R Janowska M Grivas, T. Psychological Aspects of Scoliosis Treatment in Children, Recent Advances in Scoliosis InTech 2012 DOI: 10.5772/38514 22744546

[B2] Walker C Research on Psychological Aspects of Scoliosis/Kyphosis Num 2003 15 4S 53 56

[B3] Sapountzi-Krepia D Psychogiou M Peterson D Zafiri V Iordanopoulou E Michailidou F Christodoulou A The experience of brace treatment of children/adolescents with scoliosis 2006 10.1186/1748-7161-1-8 PMC1481575 16759368

